# Carotenoid nanoforms from clementine peel stabilized by xanthan and arabic gums with antioxidant anti inflammatory and antimicrobial activities

**DOI:** 10.1038/s41598-026-45729-1

**Published:** 2026-04-14

**Authors:** Habiba A. Ahmed, Eman A. Ibrahim, Zeinab A. Salama, Sally I. Abd-El Fatah

**Affiliations:** 1 Plant Biochemistry Department, National Research Centre, Dokki, Giza, 12622 Egypt; 2 Medical Biotechnology Department, Institute of Genetic Engineering and Biotechnology, City of Scientific Research and Technological Application (SRTA-City), New Borg El-Arab, Alexandria, 21934 Egypt; 3 Food Toxicology and Contaminants Department, National Research Centre, Dokki, Giza, 12622 Egypt

**Keywords:** Clementine peels, Carotenoid nanoform, Xanthan gum, Arabic gum, Antioxidant, Anti-inflammatory, Antimicrobial activity, Biochemistry, Biological techniques, Biotechnology, Microbiology, Nanoscience and technology, Plant sciences

## Abstract

Antimicrobial resistance is a major global health threat, causing 1.27 million deaths and contributing to nearly 5 million more annually. Citrus fruits are widely cultivated for their nutritional and health benefits, and their pigments may offer additional bioactivity. This study aimed to extract carotenoids from clementine peels and quantify carotene, lycopene, astaxanthin, and anthocyanins using colorimetric methods. The carotenoids were then encapsulated using xanthan-Arabic gums matrices to stabilize the pigments. The native carotenoids and their nanoform were evaluated for antioxidants, anti-inflammatory, and antimicrobial activities. Clementine peels contained total carotenoids (30.8 mg/kg), anthocyanins (13.69 mg/kg), lycopene (3.12 mg/kg), and astaxanthin (1.60 mg/kg). The physical characterization of the carotenoid nanoparticles was performed using FTIR, confirming interactions between carotenoids and the polymer matrices. Zetasizer analysis revealed a particle size of 17.05 nm and a zeta potential of − 26.7 mV, indicating good stability, while TGA demonstrated thermal stability up to 300 °C. Antioxidant activity ranged from 18.67% to 69.10% (phosphor-molybdenum and DPPH assays). Anti-inflammatory activity showed maximum protein denaturation inhibition at 83.87% (carotenoids), 74.45% (nanoform), and 69.10% (polymers) (*p* < 0.05). Antimicrobial activity was highest against *Listeria monocytogenes* (21.3 mm) and *Penicillium verrucosum* (20 mm), moderate against *Escherichia coli* (13.0 mm) and *Aspergillus flavus* (17.0 mm), and lower against *Bacillus cereus* (10.3 mm) and *Pseudomonas aeruginosa* (9.3 mm). Overall, xanthan and Arabic gums effectively stabilized carotenoids and enhanced their biological activities.

## Introduction

Antimicrobial resistance has appeared as one of the greatest serious universal public health issues of the twenty-first period^1,2^. It occurs when microorganisms, including fungi, bacteria, and viruses, parasites, evolve to become unaffected to antimicrobial drugs, such as antibiotics, which are routinely used to treat such toxicities^3,4^. Overuse and mismanagement of antibiotics in healthcare, agricultural, and food manufacture are driving the problem worldwide^5,6^. Therefore, the global growth in antibiotic resistance and chronic inflammatory disorders has spurred interest in plant-derived bioactive compounds as potential therapeutic agents^7^.

Citrus fruits, including clementine (Citrus *Clementina*), are among the most widely cultivated fruit crops worldwide and are highly valued for their flavor, nutritional quality, and rich content of bioactive compounds^8,9^. Citrus peels are abundant sources of natural pigments, especially carotenoids such as astaxanthin, norbixin, β-carotene, lutein, bixin, lycopene, capsanthin, β-apo-8-carotenal, canthaxanthin, zeaxanthin, and β-apo-8-carotenal ester. These pigments serve dual functions: they enhance the color and sensory qualities of food products while also exhibit strong antioxidant activity as well as anti-inflammatory and anticancer properties, making them valuable candidates for nutraceutical and therapeutic applications^10–14^. However, carotenoids are highly sensitive to ecological factors such as light, temperature, oxygen, pH, low water solubility, and quick degradation in the gastrointestinal system limit their practical applicability^15,16^. Humans are unable to synthesize carotenoids and therefore must obtain them through dietary sources or supplements. Therefore, current research focuses on nanoencapsulation, which has emerged as a promising method for increasing hydrophobic bioactive stability, bioavailability, and controlled release^17^. Natural biopolymers, such as Xanthan and Arabic gums, are excellent stabilizers due to their biocompatibility, emulsifying capabilities, and ability to build protective matrices around nanoparticles^18^. Xanthan gum, a microbial polysaccharide, improves colloidal stability^19^, whereas Arabic gum, a plant exudate, increases encapsulation efficiency^20^. Encapsulation techniques, including spray drying with polysaccharides, have emerged as effective strategies to improve the stability and bioavailability of carotenoids^21,22^. The novelty of the present work is a combined strategy that integrates carotenoid extraction from clementine peel with their encapsulation in Arabic-xanthan gums biopolymer composite matrix. This approach enhances the physicochemical properties and functional performance of the extracted carotenoids, aspects that have received limited attention in earlier reports. In addition, the antioxidant, antimicrobial, and anti-inflammatory activities of the encapsulated carotenoids were systematically evaluated, providing a scientific basis for selecting the most suitable applications in future food and pharmaceutical studies.

## Materials and methods

### Chemicals

Ethanol, hexane, acetone, and petroleum ether were of high grade and obtained from Merck KGaA (Darmstadt, Germany). Potato dextrose agar and nutrient agar were obtained from Neogen (Lansing, MI 48,912, USA). Dimethyl sulfoxide (DMSO) was purchased from the Research Lab Fine Chem Industries (India). Ascorbic acid, sulfuric acid, hydrochloric acid, and sodium phosphate were obtained from Merck KGaA (Darmstadt, Germany). The 1,1-diphenyl-2-picryl hydrazyl (DPPH), ammonium molybdate, xanthan gum, and Arabic gum were purchased from Sigma Aldrich (St Louis, MO, USA).

### Microorganisms

Three gram-positive pathogenic bacteria (*Bacillus cereus* EMCC1080, *Staphylococcus aureus* ATCC13565, and *Listeria monocytogenes*) and three Gram-negative bacteria (*Salmonella typhi* ATCC15566, *Escherichia coli* O157-H7ATCC51659, and *Pseudomonas aeruginosa* NRRLB-272) were kindly provided from the collection of the Dairy Microbiological Lab, National Research Centre, Egypt. Seven fungal species (*Candida albicans* ATCC 10231, *Aspergillus flavus* NRRL 3357, *Aspergillus ochraceus* ITAL 14, *Aspergillus niger* ATCC- 16888, *Fusarium proliferatum* MPVP328, *Fusarium verticillioides* ITEM 10027 and *Penicillium verrecosum* ITEM10027) were obtained from Food Toxicology and Contaminants Dept. National research Center.

### Preparation of clementine peels

Fresh clementine fruits were obtained from a local market in Cairo, Egypt. The peels were washed and dried for 48 h at 35 °C in a laboratory oven (Heraeus T 5050 E, 250 °C). After drying, the samples were ground using an electric grinder and passed through a conventional 500 μm sieve.

### Extraction of pigments from clementine peel

Carotenoids (pigments) were extracted from dried clementine peels using a solvent extraction method. Briefly, 100 g of dried peel powder was mixed with 100 mL of an ethanol/hexane solvent mixture (1:1, v/v) and shaking for 24 h at room temperature. The resulting extract was filtered, and the solvents were evaporated under reduced pressure to obtain the crude pigment extract^23^.

### Percent yield of extract

The extraction yield was calculated using the following equation

$$\:\mathrm{Extraction} \:\:\mathrm{yield}\:\left(\mathrm{\%}\right)=\left(\frac{\mathrm{M}\mathrm{a}\mathrm{s}\mathrm{s}\:\:\mathrm{o}\mathrm{f}\:\:\mathrm{e}\mathrm{x}\mathrm{t}\mathrm{r}\mathrm{a}\mathrm{c}\mathrm{t}\:\left(\mathrm{g}\right)}{\mathrm{M}\mathrm{a}\mathrm{s}\mathrm{s}\:\:\mathrm{o}\mathrm{f}\:\:\mathrm{d}\mathrm{r}\mathrm{i}\mathrm{e}\mathrm{d}\:\:\mathrm{p}\mathrm{e}\mathrm{e}\mathrm{l}\:\:\mathrm{p}\mathrm{o}\mathrm{w}\mathrm{d}\mathrm{e}\mathrm{r}\:\left(\mathrm{g}\right)}\right)\times\:\left(100\right)$$.

### Identification of pigments in the crude extract by spectrophotometry

1. Determination of total carotenoids

Total carotenoids were measured according to a previously described method^24^, with minor modifications. One gram of dried crude clementine extract was mixed with 20 mL of acetone. The mixture was incubated in a water bath at 50 °C for 90 min. After incubation, the extract was filtered through filter paper, and the absorbance of the filtrate was measured at 445 nm using a UV-visible spectrophotometer. The quantity of carotenoids was determined by the equation below

$$\:\mathrm{T}\mathrm{o}\mathrm{t}\mathrm{a}\mathrm{l}\:\:\mathrm{c}\mathrm{a}\mathrm{r}\mathrm{o}\mathrm{t}\mathrm{e}\mathrm{n}\mathrm{o}\mathrm{i}\mathrm{d}\:\left(\frac{\mathrm{m}\mathrm{g}}{100\mathrm{g}}\right)=\raisebox{1ex}{$\mathrm{A}\:\times\:\mathrm{V}\left(\mathrm{m}\mathrm{l}\right)$}\!\left/\:\!\raisebox{-1ex}{${10}^{6}\:\mathrm{A}1\:\mathrm{c}\mathrm{m}\mathrm{\%}\:\times\:{10}^{3}\:\mathrm{A}1\:\mathrm{W}$}\right.$$

where A is the absorbance of the extract at 445 nm. V is the extract volume (mL). W is the weight of the ground clementine peel (g). %A_1 cm_ is the specific extinction coefficient of carotenoids (2559 for acetone).

2. Determination of total astaxanthin.

The total astaxanthin content was determined according to a previously described method^25^. A 0.5 mg sample of clementine peel crude extract was combined with 2 mL of dimethyl sulfoxide. After standing for 5 min, the sample was incubated at 70 °C and measured the absorbance at 492 nm. The results were expressed as mg astaxanthin equivalents per gram of dry extract. 

$$\:\mathrm{T}\mathrm{o}\mathrm{t}\mathrm{a}\mathrm{l}\:\:\mathrm{a}\mathrm{s}\mathrm{t}\mathrm{a}\mathrm{x}\mathrm{a}\mathrm{n}\mathrm{t}\mathrm{h}\mathrm{i}\mathrm{n}\:\:(\mathrm{m}\mathrm{g}/\mathrm{g}\:\:\mathrm{d}\mathrm{r}\mathrm{y}\:\:\mathrm{e}\mathrm{x}\mathrm{t}\mathrm{r}\mathrm{a}\mathrm{c}\mathrm{t})\:=\left(\frac{\mathrm{C}\times\:\mathrm{V}}{\mathrm{m}}\right)$$

where $\:\mathrm{C}$ is the astaxanthin concentration determined from the standard curve (mg/mL). $\:\mathrm{V}$ is the volume of extract (mL). $\:\mathrm{m}$ is the mass of dry extract used (g).

3. Determination of total anthocyanins.

The determination of total anthocyanins was carried out as previously described^26^, with some modifications. The absorbance of the clementine peel crude extract was measured at 540 nm after dilution with a mixed solvent (70:29:1) of ethanol, water, and HCl. Total anthocyanin content was calculated in malvidin-3-glucoside equivalents, using the following formula. 

$$\:\mathrm{T}\mathrm{A}=\mathrm{D}\mathrm{i}\mathrm{l}\mathrm{u}\mathrm{t}\mathrm{i}\mathrm{o}\mathrm{n}\:\:\mathrm{o}\mathrm{f}\:\:\mathrm{s}\mathrm{a}\mathrm{m}\mathrm{p}\mathrm{l}\mathrm{e}\times\:16.7\times\:\mathrm{d}$$

where TA is the total anthocyanin content, D is the dilution factor. 16.7: It is a constant (conversion factor) that comes from the relationship between volume of NaOH used, its normality (usually 0.1 N), and the equivalent weight of the acid.

4. Determination of lycopene content.

The lycopene content in clementine crude extract was determined using a hexane-acetone mixture^27^. Then, the extract was passed through paper filters, and measured absorbance at 472 nm. The lycopene content in the clementine extract (w, mg/kg) can be calculated using following equation.

$$\mathrm{w}=\raisebox{1ex}{$\mathrm{A}\mathrm{*}\mathrm{V}\mathrm{*}\mathrm{D}{10}^{4}$}\!\left/\:\!\raisebox{-1ex}{$E*l*m$}\right.$$

In this equation, A represents the absorbance of the diluted extract at 472 nm.

V is the volume of the single-marked volumetric flask used for extraction (50 mL).

D denotes the dilution ratio of the sample.

E is the specific absorbance (molar extinction coefficient) of lycopene in petroleum ether (3450 for 1% 1 cm).

L represents the optical path length of the absorption cell in cm; and m is the mass of the sample.

### Preparation of carotenoid nanoparticles

Carotenoid nanoparticles were prepared following a previously described method^28^, with minor modifications^28^. Briefly, carotenoids (0.3 g) were dissolved in 25 mL of distilled water containing 3 mL of Tween 80 and stirred for 2 h. Xanthan gum (0.5 g) was separately dissolved in 150 mL of distilled water and stirred for 2 h, while Arabic gum (1 g) was dissolved in 50 mL of distilled water and stirred for 2 h. The Arabic gum solution was then added dropwise to the xanthan gum solution under continuous stirring. Subsequently, the carotenoid solution was added dropwise to the mixture with constant stirring. The resulting solution was sonicated using an ultrasonic processor (DAIGGER Ultra-Sonic Model GEX 750, USA) for 15 min. Finally, the suspension was freeze-dried and stored at − 20 °C in an airtight container until use.

### Physiochemical characterization

#### Fourier transform infrared spectroscopy (FTIR)

Fourier transform infrared (FTIR) spectra were recorded using a Bruker Optics Tensor 27 spectrometer (Bruker Corporation, Billerica, MA, USA) to investigate functional groups and intermolecular interactions between carotenoids and the encapsulating materials (xanthan gum, Arabic gum and tween 80). Measurements were performed in transmission mode within the spectral range of 4000–400 cm^−1^ at a resolution of 4 cm^−1^^29^.

#### Thermogravimetric analysis (TGA)

The thermal stability of carotenoids and their nano-formulations was assessed by thermogravimetric analysis using a SETARAM Themys One Plus analyzer (France). Samples (~ 10 mg) were heated from 25 °C to 700 °C at a constant rate of 10 °C/min under a nitrogen atmosphere (flow rate: 50 mL/min) to prevent oxidative degradation. Data was analyzed using Calisto Processing Software to determine decomposition temperatures and residual mass percentages.

#### Determination of particle size, PDI, and zeta potential

The characteristics of nanoparticles, including zeta potential, PDI, and size distribution were analyzed in aqueous suspension (25 °C) using dynamic light scattering (DLS) on a Malvern Zetasizer Nano ZS ^30^.

### Antioxidant activity

#### DPPH radical scavenging activity

Free radical scavenging activity was determined using the 2,2-diphenyl-1-picrylhydrazyl (DPPH) assay^31^. Samples at different concentrations (50, 100, and 150 µg/mL) were mixed with 3.0 mL of 0.1 mM DPPH solution in ethanol. After incubation for 30 min in complete darkness, absorbance was measured at 517 nm using a UV–visible spectrophotometer. A blank containing ethanol instead of DPPH and a control containing DPPH without sample were prepared under the same conditions. Ascorbic acid (vitamin C) was used as a positive control. The percentage of DPPH radical scavenging activity was calculated using following Equation.

$$\:\mathrm{S}\mathrm{c}\mathrm{a}\mathrm{v}\mathrm{e}\mathrm{n}\mathrm{g}\mathrm{i}\mathrm{n}\mathrm{g}\mathrm{\%}=\left[\raisebox{1ex}{$1-({A}_{sample}-{A}_{blank}$}\!\left/\:\!\raisebox{-1ex}{${A}_{control}$}\right.)\right]\times\:100$$

#### Total antioxidant capacity (phosphor-molybdenum)

Total antioxidant capacity (TAC) was measured using the phosphor-molybdenum reduction assay^32^. The reagent solution was prepared by mixing 1 mL of 0.6 M sulfuric acid, 28 mM sodium phosphate, and 4 mM ammonium molybdate with distilled water and adjusting the final volume to 50 mL. For the assay, 0.3 mL of extract or standard at different concentrations (50, 100, and 150 µg/mL) was added to 3 mL of the reagent solution. The tubes were incubated at 95 °C for 90 min, then cooled to room temperature. Absorbance was measured at 695 nm using a UV-visible spectrophotometer against a blank containing ethanol and reagent. Ascorbic acid was used to prepare a standard curve, and TAC was expressed as µg ascorbic acid equivalents (AAE) per mg of extract, calculated using the following equation: 

$$\:\mathrm{T}\mathrm{A}\mathrm{C}\:\:({\upmu\:}\mathrm{g}\:\:\mathrm{A}\mathrm{A}\mathrm{E}/\mathrm{m}\mathrm{g}\:\:\mathrm{e}\mathrm{x}\mathrm{t}\mathrm{r}\mathrm{a}\mathrm{c}\mathrm{t})=\left(\frac{C\times\:V}{M}\right)$$

where $\:C$ is the concentration of ascorbic acid equivalent obtained from the standard curve (µg/mL). $\:V$ is the volume of the extract used in the assay (mL). $\:M$ is the mass of the extract (mg).

#### Anti-inflammatory activity

The in vitro anti-inflammatory activity was evaluated using the bovine serum albumin (BSA) protein denaturation assay^33^, with slightly modifications. Briefly, 0.5 mL of 0.2% BSA solution was mixed with samples or standard (diclofenac sodium) at concentrations of 50, 100, and 150 µg/mL and incubated at 37 °C for 20 min. The mixture was then heated at 72 °C for 5 min to induce protein denaturation, cooled to room temperature, and 2.5 mL of phosphate buffer (pH 6.3) was added. Absorbance was measured at 660 nm using a UV–visible spectrophotometer against a blank containing BSA and buffer. The percentage of protein denaturation inhibition was calculated using following Equation:

1 $${\rm{Inhibition }}\left( \% \right){\rm{ }} = {\rm{ }}\left[ {\left( {{\rm{Ab}}{{\rm{s}}_{{\rm{control}}}}{-}{\rm{Ab}}{{\rm{s}}_{{\rm{sample}}}}} \right){\rm{ }}/{\rm{ Ab}}{{\rm{s}}_{{\rm{control}}}}} \right]{\rm{ }} \times {\rm{ 1}}00$$

#### Antimicrobial activity

The microbial inhibition activity of Arabic gum, xanthan gum, clementine peel carotenoids, and carotenoid nanoparticles (NPs) was determined using the disk diffusion method^34^. The bacterial strains were grown on nutrient agar dishes at 37 °C for 24 h. The fungal strains were inoculated on potato dextrose agar for 7 days at 28 °C. The tested microorganisms were inoculated into Tryptic Soy broth tubes and incubated at 37 °C for 4 h, and the turbidity of these cultures was adjusted using 0.5 McFarland. Sterile cotton swabs developed a uniform bacterial lawn on the surface of solid nutrient agar plates. Fungal spores were mixed in saline solution with 0.01% of Tween 80 and spread on potato dextrose agar plates. The filter paper No.1 discs (6 mm), which were soaked in Arabic gum, xanthan gum, carotenoids of clementine peel, and carotenoid-NPs (100 mg/mL in 5% DMSO). The impregnated discs were applied to the surface of streaked nutrient agar and potato dextrose agar plates. The nutrient agar plates were incubated at 35 °C for 16-18 h, whereas the potato dextrose agar plates were incubated at 28 °C for 24 to 48 h. Ceftriaxone (1 mg/mL) was used as an antibacterial agent, and miconazole (1 mg/mL) was used as an antifungal agent. The inhibition zones were measured to the nearest millimeter using a ruler.

### Statistical analysis

All experiments were performed in triplicate, and results are presented as mean ± standard deviation (SD). Statistical comparisons among groups were performed using one-way analysis of variance (ANOVA) with CoStat for Windows (version 6.45 Differences were considered statistically significant at a significance level of *p* < 0.05^35^.

## Results and discussion

### Yield of crude pigments

The extraction of crude pigments from dried clementine peels resulted in a yield of 18 g per 100 g of dry weight, indicating that the solvent system effectively recovered the carotenoids. This suggests that clementine peels are a promising source of natural pigments.

### The pigments content of clementine peels

Data presented in Table 1 showed that carotenoids were the most prevalent pigments in the peels (30.8 mg/kg), followed by total anthocyanins (13.69 mg/kg), while lycopene and astaxanthin were present at lower levels of 3.12 and 1.6 mg/kg, respectively. Citrus peels are rich in natural pigments, including fat-soluble carotenoids and water-soluble pigments such as anthocyanins, which contribute to their characteristic coloration^36^. These results are consistent with a previous study^37^, showing that carotenoids were the major pigments in clementine peels (8.01 µg/mL), followed by anthocyanins (3.17 µg/mL) and astaxanthin (0.4 µg/mL), with all three detected at higher concentrations in the present study. These variations may be attributed to differences in citrus species, peel maturity, environmental conditions, and extraction or analytical methods. Overall, the lower levels of lycopene and astaxanthin indicate that these pigments are minor constituents of citrus peels compared to carotenoids and anthocyanins.

**Table 1:** Pigments of citrus clementine peels.

Pigments	Concentration (mg/kg dry peel)
Total carotenoids	30.81^a^ ± 0.015
Total astaxanthin	1.61^d^ ± 0.017
Total anthocyanin	13.68^b^ ± 0.01
Lycopene	3.11^c^ ± 0.05
LSD	0.024

Results are mean ± SD (*n* = 3).

Within each column, different letter subscripts are considered significant (*P* < 0.05).

### Physicochemical characterization of nanoparticles

#### Fourier-transform infrared spectroscopy (FTIR)

**Fig. 1:**
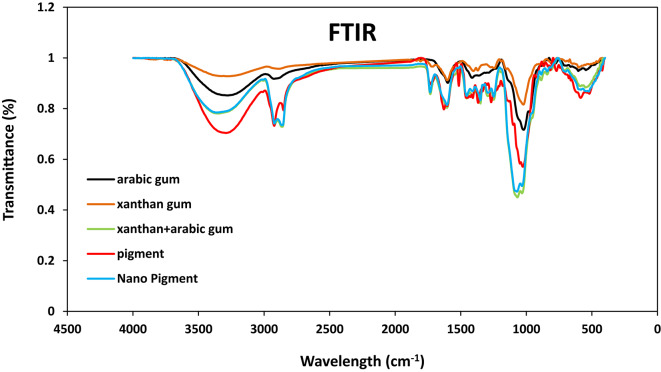
Fourier-transform infrared spectroscopy spectra of Arabic gum (black), xanthan gum (orange), Arabic and xanthan gum (green), carotenoids (red), and nano-carotenoids (blue).

Data in Fig. 1 displayed the peaks that showed the complicated nature of the Arabic gum with distinct peaks at 3274 cm^1^ (corresponding to the –OH group), 2925 cm^1^ (corresponding to –CH2), 1599 cm^1^ (corresponding to –C–O), 1413 cm^1^ (corresponding to –CH), 1020 cm^1^ (assigned to C–O–C), and 603 cm^1^ (indicating a –C–H linkage). The findings agree with those reported previously^38^. The xanthan gum exhibited absorption bands at 3282 cm^1^ (O–H axial deformation), 2894 cm^1^ (C–H bonds stretching vibrations in methyl and methylene groups), 1712 cm^1^ (C=O stretching vibrations), and 1603 cm^1^ (axial deformation of the C–O part of the enols)^39^. The mixtures of Arabic and xanthan gums showed distinct peaks at 3343, 2920, 2859, 1732, 1605 cm^1^, 1454 cm^1^, 1411 cm^1^, 1350 cm^1^, 1293 cm^1^, 1284 cm^1^, 1067 cm^1^, 886 cm^1^, and 547 cm^1^, respectively. The data revealed a high overlap of peaks from Arabic and xanthan gums, suggesting significant interactions between the two polymers. The native carotenoid pigments showed strong absorption bands at 3290 cm^1^ (assigned to the O–H group), 2922 cm^1^ (–CH2), 1733 cm^1^ and 1628 cm^1^ (attributed to C = O and C = C groups, respectively, in the aromatic rings), 1514 cm^1^ and 1407 cm^1^ (corresponding to C–O vibrations), 1268 cm^1^ (assigned to the stretching of the pyran rings), 1027 cm^1^ (owing to the stretching vibration of C–O–C esters), and 585 cm^1^ (indicating the presence of aromatic rings)^40^. Therefore, spectral analysis of the concentrated extracts shows the presence of functional groups such as –OH, C=O, C=C, and C–O–C, which are characteristic of anthocyanins^41^. The nano-carotenoids showed distinct peaks at 3367 cm^1^, 2921 cm^1^, 2859 cm^1^, 1733 cm^1^, 1604 cm^1^, 1514 cm^1^, 1407 cm^1^, 1268 cm^1^, 1071 cm^1^, and 585 cm^1^. FTIR spectra confirmed successful loading of carotenoids into the Arabic/xanthan gum nanoparticle matrix, primarily via hydrogen bonding and pH-sensitive ionic interactions, with minimal chemical modification as indicated by preserved characteristic bands^42^.

#### Thermogravimetric analysis

**Fig. 2:**
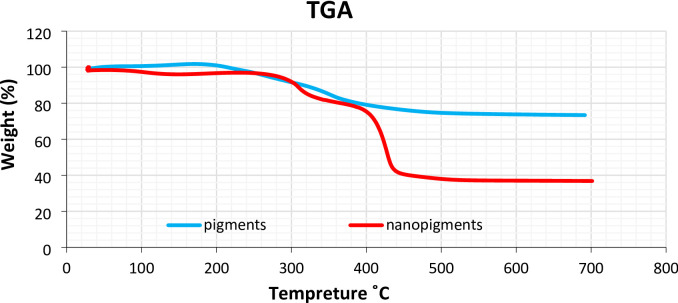
Thermogravimetric analysis of carotenoids (pigments) and their nano-form (nano pigments).

The thermal stability of carotenoids and their nanoform is presented in Fig. 2. Carotenoids exhibited an initial weight loss of approximately 10% between 300 °C and 316 °C, followed by a further loss of up to 27% between 563 °C and 691 °C. When carotenoids were converted into nanoparticles, a 10% weight loss between 303 °C and 306 °C, followed by a 27% weight loss between 404 °C and 406 °C. The early weight loss in the second stage of the nanoform is likely due to moisture evaporation and degradation of the polymeric matrix, while the overall structure remains stable up to approximately 306 °C.

#### Particle size distribution

**Fig. 3:**
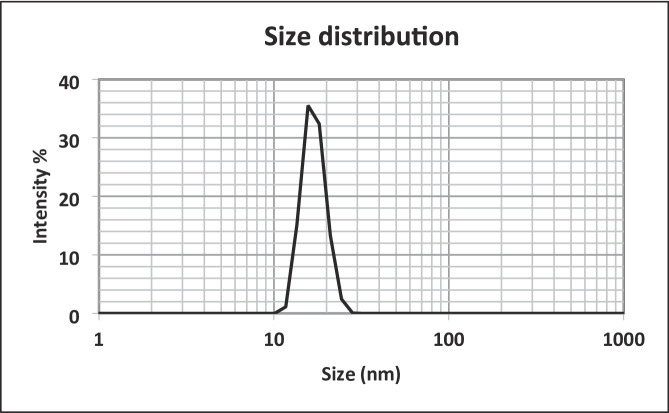
Particle size distribution of carotenoids-NPs.

The surface charge (zeta potential), polydispersity index (PDI), and particle size are critical factors influencing the stability and effectiveness of nanomedicine in drug delivery. Figure 3 shows that the produced carotenoid nanoparticles have a size of 17.05 ± 2.59 nm, confirming they are within the nanoscale range. Data in Table 2 showed that the surface charge of the carotenoids NPs was − 26.7 mV, which is higher than + 5 mV, suggesting a low tendency for coagulation or flocculation of the produced carotenoid NPs. Although The polydispersity index (PDI) value of 0.55 indicates moderate size heterogeneity, this level of distribution is still acceptable for functional performance. Nevertheless, achieving a lower PDI (≤ 0.5) through formulation optimization could enhance particle uniformity, reproducibility, and long-term stability, which is an important consideration for future studies^30^.

**Table 2:** Zeta potential and polydispersity index (PDI) of carotenoid-NPs.

Parameters	Carotenoid-NPs
Zeta potential (mv)	− 26.7
PDI	0.55

#### Antioxidant activity

1. DPPH assay

The antioxidant activity of clementine peel carotenoids, their nanoparticles, Arabic gum, xanthan gum, and their combination is summarized in Table 3. The DPPH· scavenging activity ranged from 18.67% for xanthan–Arabic gums at 25 µg/mL to 69.1% for free carotenoids at 75 µg/mL compared to the control. Interestingly, the combination of Arabic-xanthan gums (66.44%) and the individual gums (xanthan 64.79%, Arabic 61.49%) also demonstrated notable antioxidant activity, suggesting that these polysaccharides may contribute through hydrogen-donating or stabilizing mechanisms. Carotenoid nanoparticles showed slightly lower inhibition (61.07%) than free carotenoids. This decrease can be attributed to the partial encapsulation of carotenoids within the nanoparticle matrix, which restricts their immediate interaction with free radicals. As a result, the antioxidant molecules are released gradually rather than acting all at once, leading to a lower short-term inhibition in assay^43^. Despite this, both free and nanoform carotenoids, as well as gum-based formulations, demonstrated significant antioxidant activity, highlighting their potential for food and biomedical applications.

2. Phospho-molybdenum assay

The phospho-molybdenum assay evaluates the total antioxidant capacity (TAC) based on the reduction of Mo (VI) to Mo (V) by antioxidants under acidic conditions, resulting in the formation of a green phosphate/Mo(V) complex measurable at 695 nm. The extent of reduction reflects the electron-donating ability of the tested compounds. As shown in Table 3, all tested samples exhibited notable antioxidant activity, though to varying degrees. The free carotenoids displayed the highest activity among the treatments (40.52%, 43.01%, and 47.75% at 50, 100, and 150 µg/mL, respectively), comparable to the vitamin C standard. This strong activity is likely due to the conjugated double-bond system of carotenoids, which effectively donate electrons to reduce Mo (VI) and neutralize reactive species. Carotenoid nanoparticles exhibited lower total antioxidant capacity (TAC) values (25.23–29.54%) compared to free carotenoids. This reduction is likely due to the partial entrapment of carotenoids within the polymeric matrix, which limits their immediate availability in the assay and delays release. Notably, the main advantage of nanoencapsulation is not an enhancement of short-term antioxidant activity, but rather the protection of carotenoids from environmental degradation (e.g., light, oxygen, and heat) and the facilitation of controlled release. This sustained release can improve stability, preserve bioactivity over time, and extend antioxidant effects in practical applications^43^. Polymers such as xanthan and Arabic gums showed moderate antioxidant capacities (31–38%), likely associated with their hydroxyl functional groups capable of mild reducing activity. Their combination (xanthan-Arabic gums) yielded slightly enhanced inhibition at higher concentrations, indicating potential synergistic stabilization of carotenoids in a mixed polymer matrix. These results agree with earlier studies^43,44^, showing strong antioxidant activity of carotenoid extracts (IC_50_ = 0.14 mg/mL) and improvement after nanoencapsulation. The present results confirm that carotenoids are the primary contributors to the total antioxidant capacity of clementine peel extracts, acting mainly through electron transfer and radical neutralization mechanisms.

**Table 3:** DPPH and phosphomolybdic acid scavenging (%) by nano-formulated carotenoid and polymers.

Treatments	Concentration (µg/ml)		
	25	50	75
DPPH			
(Vit.C)	25.83^b^ ± 0.03	28.38^d^ ± 0.11	40.87^e^ ± 0.03
Carotenoid	30.17^a^ ± 0.06	40.52^a^ ± 0.24	69.10^a^ ± 0.11
Carotenoid-NPs	20.32^d^ ± 0.03	25.23^e^ ± 0.12	61.07^d^ ± 0.76
Xanthan Gum	22.27^c^ ± 0.06	33.31^b^ ± 0.29	64.79^c^ ± 0.54
Arabic Gum	19.55^e^ ± 0.05	31.21^c^ ± 0.06	61.49^d^ ± 0.11
Xanthan + Arabic Gum	18.67^f^ ± 0.04	24.18^f^ ± 0.02	66.44^b^ ± 0.05
LSD	0.08	0.29	0.68
Phosphomolybdic acid			
(Vit.C)	28.38^d^ ± 0.11	42.53^b^ ± 0.02	50.29^a^ ± 0.06
Carotenoid	40.52^a^ ± 0.24	43.01^a^ ± 0.12	47.75^b^ ± 0.12
Carotenoid-NPs	25.23^e^ ± 0.12	26.65^f^ ± 0.12	29.54^f^ ± 0.14
Xanthan Gum	33.31^b^ ± 0.29	34.86^c^ ± 0.05	38.38^c^ ± 0.03
Arabic Gum	31.21^c^ ± 0.06	33.19^d^ ± 0.02	37.07^d^ ± 0.04
Xanthan + Arabic Gum	24.18^f^ ± 0.02	28.17^e^ ± 0.06	32.34^e^ ± 0.05
LSD	0.29	0.13	0.14

Results are mean ± SD (*n* = 3).

Within each column, different letter subscripts are considered significant (*P* < 0.05).

### Anti-inflammatory

Inflammation is often driven by protein denaturation, making inhibition of this process a key indicator of anti-inflammatory potential. As shown in Table 4, carotenoids pigments exhibited the highest anti-inflammatory activity, with inhibition values of 69.10%, 74.45%, and 83.87% (at 50, 100 and 150 µg/mL respectively), while nano-formulated carotenoids (Carotenoid-NPs) and polymeric matrices (xanthan and Arabic gums) demonstrated moderate effects compared to the control (diclofenac sodium). The potent activity of carotenoids can be attributed to their lipid-soluble nature and previously reported anti-inflammatory properties in both in vitro and in vivo studies^45^. The lower short-term anti-inflammatory activity of Carotenoid-NPs is likely due to the partial entrapment of carotenoids within the polymeric matrix, which restricts their immediate availability to interact with inflammatory mediators. However, this gradual release can be advantageous, as it protects the active compounds and may maintain anti-inflammatory effects over an extended period^16^. Anthocyanin-rich fraction was found to inhibit NF-κB activation elicited by IL-1β in intestinal epithelial Caco-2 cells, whereas the doses of 50 and 100 µg/mL reduced NF-κB activation by 68.9 and 85.2%, respectively^46^. Prior study showed significant changes in the anti-inflammatory activity of anthocyanin at different concentrations (50–150 µg/mL), whereas the maximum inhibition record was 52.13%, which was seen from anthocyanin at 150 µg/mL. Lycopene has been demonstrated to reduce the progress of the TLR4/adaptor complex on the membrane, which could be attributed to decreased TLR4 translocation to the lipid raft^47^. Lycopene may interfere with TLR4’s connection with adaptors, resulting in TLR4 deactivation, and dramatically inhibiting the stimulation of NF-κB and production of NO/IL-6 produced by LPS^48^. Additionally, it lowered the formation of the TLR4 complex with MyD88 or TRIF. Furthermore, gold nanoparticles synthesized from *Citrus sinensis* fruit peel extract effectively stopped heat-induced albumin denaturation. At a concentration of 50 µg/mL, the highest inhibition of 76.64% was observed, indicating that gold nanoparticles can prevent protein denaturation, which contributes to inflammation^49,50^.

**Table 4:** Anti-inflammatory activity of carotenoids and nano-encapsulated formulations.

Treatments	Percentage of inhibition (%)		
	50 µg/mL	100 µg/mL	150 µg/mL
Control (diclofenac sodium)	40.87^e^ ± 0.03	61.00^f^ ± 0.01	80.70^b^ ± 0.00
Carotenoid	69.10^a^ ± 0.11	74.45^a^ ± 0.14	83.87^a^ ± 0.42
Carotenoid-NPs	61.07^d^ ± 0.76	65.15^d^ ± 0.13	69.62^d^ ± 0.02
Xanthan Gum	64.79^c^ ± 0.54	66.17^c^ ± 0.66	67.23^e^ ± 0.02
Arabic Gum	61.49^d^ ± 0.11	62.00^e^ ± 0.00	64.38^f^ ± 0.16
Xanthan + Arabic Gum	66.44^b^ ± 0.05	69.24^b^ ± 0.05	70.08^c^ ± 0.02
LSD	0.68	0.50	0.32

Results are mean ± SD (*n* = 3).

Within each column, different letter subscripts are considered significant (*P* < 0.05).

### Antimicrobial activity

The antibacterial activity of carotenoid pigments of clementine peels and their nanoform compared to Arabic gum, xanthan gum, and the mixture of xanthan and Arabic gums was studied. Data in Table 5 showed that the mixture of xanthan and Arabic gums inhibited *B. cereus*, recording a zone inhibition of 10.3 mm, followed by carotenoid pigments, recording a zone inhibition of 8.3 mm, respectively. Concerning *S. aureus*, results indicated that carotenoid pigments and its NPs inhibited *S. aureus*, recording an inhibition zone of 9.0 and 8.7 mm, respectively. Both carotenoid pigments and Arabic gum inhibited *L. monocytogenes*, recording a zone inhibition of 21.3 and 21.0 mm, respectively. Arabic gum also inhibited *E. coli* and *S. typhi*, causing a zone of inhibition of 13.0 and 12.7 mm, respectively. Carotenoid pigments also inhibited *E. coli* and *S. typhi*, showing a zone of inhibition of 10.0 and 11.0 mm, respectively. A mixture of Xanthan and Arabic gums revealed a zone of inhibition of *P. aeruginosa* of 9.3 mm, followed by Arabic gum alone, which inhibited *P. aeruginosa* by 9.3 mm. The positive control, ceftriaxone, displayed a significant difference (*P* < 0.05) from carotenoid pigments, its NPs, and polymers used for all bacteria studied. The moderate efficacy of free carotenoids and their nanoform suggests their potential as natural antimicrobial agents, particularly in food preservation or biomedical applications where controlled release and biocompatibility are advantageous. These results also support the idea that nanoencapsulation can maintain bioactivity while offering additional benefits such as protection from degradation and sustained release over time^22^.

The antifungal activity of carotenoid pigments, their nanoform, and polysaccharide matrices (Arabic and xanthan gums) was evaluated against several fungal species (Table 6). The mixture of xanthan and Arabic gums inhibited *C. albicans*, showing a zone of inhibition recording 12.0 mm, followed by carotenoid pigments, which inhibited *C. albicans* by a zone of inhibition recording 9.7 mm. Xanthan gum showed high antifungal activity and inhibited *A. flavus* with a zone of inhibition displaying 17.0 mm. Xanthan gum alone, and a mixture of xanthan and Arabic gum, inhibited *A. niger* with a zone of inhibition recording 11.7 and 10.0 mm correspondingly. Meanwhile, carotenoid pigments and xanthan gum showed a similar zone of inhibition for *A. ochraceus*, recording 10.0 mm. The fungi *P. verrecosum* was inhibited by Arabic gum and a mixture of xanthan and Arabic gum, displaying a zone of inhibition of 20.0 and 18.3 mm, correspondingly. Arabic gum alone inhibited *F. verticillioides* and exhibited a zone of inhibition of 15.7 mm. Meanwhile, *F. oxysporum* was inhibited by carotenoid NPs, showing a zone of inhibition recording 14.7 mm. The positive control, miconazole, demonstrated a significant difference (*P* < 0.05) from carotenoid pigments, its NPs, and polymers used for all fungi studied except *F*. *verticillioides* and *F. oxysporum*.

According to reports, Arabic gum has been found to have inhibitory properties against *S. typhi*, *B. cereus*, *S. aureus*, *E. coli*, and even *C. albicans*, and *A. niger*^51,52^. Arabic gum has been successfully employed as a carrier of other antimicrobial herbal preparations (garlic extract or cinnamon) to build an antibacterial coating on foods, even though its antimicrobial activity is rarely observed. Xanthan-oligosaccharide had antibacterial action against *S. aureus*, whereas the mechanism of action indicated that it not only reduced cell membrane permeability but also prevented biofilm development and Ca_2_^+^–Mg_2_^+^-ATPase activity on the *S. aureus* cytomembrane^53^. The previous study showed that the citrus peel and carotenoids have a high antimicrobial effect against different bacteria and fungi. The susceptibility of gram-positive bacteria to these pigments, nano-formulations, and polymer gums can be attributed to the bacteria’s thick hydrophobic cell wall structure, which facilitates interaction with these compounds^54,55^. Also, the pigments carotenoids showed strong effect on *S. aureus*^56^.

Food safety is a major concern in modern food production, as contamination by pathogens and spoilage microorganisms can pose substantial health hazards. This shows that adding carotenoids to food packaging or preservation methods could aid in decreasing microbial contamination and increasing shelf life^57,58^. The usage of natural compounds for food preservation is gaining popularity as customers seek alternatives to synthetic additives. Clementine peel carotenoids stabilized with xanthan and Arabic gums increase their bioavailability and efficiency, making them a promising natural preservative.

**Table 5:**
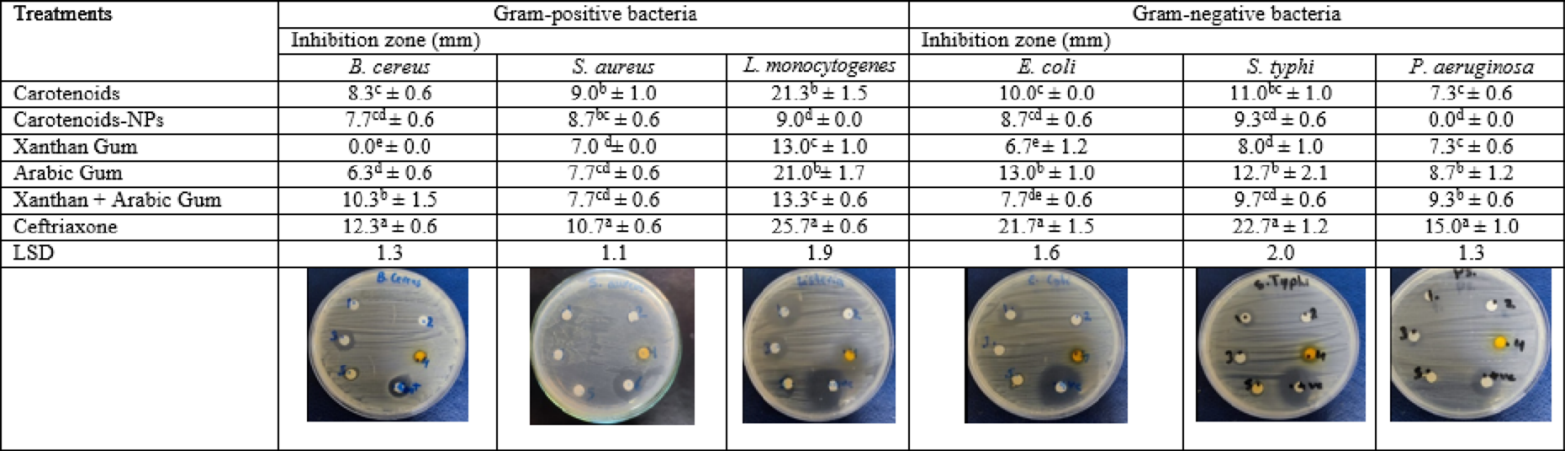
Antibacterial activity of nano-formulated carotenoids and polymers at 100 mg/mL.

Results are mean ± SD (*n* = 3).

Within each column, different letter subscripts are considered significant (*P* < 0.05).

**Table 6:**
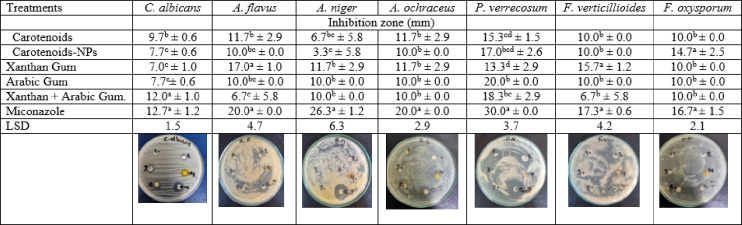
Antifungal activity of nano-formulated carotenoids and polymers at 100 mg/mL.

Results are mean ± SD (*n* = 3).

Within each column, different letter subscripts are considered significant (*P* < 0.05).

## Conclusion

Carotenoids nanoform from clementine peels stabilized with xanthan and Arabic gums represent a promising advancement in food safety and pharmaceuticals. Their antioxidants, antimicrobial, and anti-inflammatory properties suggest potential applications such as natural food preservatives or antibiotic formulations. However, the antimicrobial findings are preliminary and require further quantitative evaluation, such as MIC determination and mechanistic studies, to confirm efficacy. The combined use of these gums enhances both stability and bioactivity, offering opportunities in functional foods and natural health products. While this study provides preliminary insights into their therapeutic potential, further research is needed to establish safety and efficacy. Future work will focus on determining cytotoxicity and gastrointestinal stability assessments, and performing synergistic analyses, such as checkerboard and fractional inhibitory concentration index (FICI) tests, to fully evaluate their application in food and pharmaceutical products.

## Data Availability

All data generated or analyzed during this study are included in this published article.
